# Proteomic analysis of zebrafish (*Danio rerio*) embryos exposed to benzyl benzoate

**DOI:** 10.1007/s11356-022-24081-7

**Published:** 2022-11-11

**Authors:** Young Sang Kwon, Chang-Beom Park, Seung-Min Lee, Seonggeun Zee, Go-Eun Kim, Yeong-Jin Kim, Hee-Jung Sim, Jong-Hwan Kim, Jong-Su Seo

**Affiliations:** 1 Environmental Safety Assessment Center, Gyeongnam Branch Institute, Korea Institute of Toxicology, Jinju, 52834 Republic of Korea; 2 Environmental Exposure and Toxicology Center, Gyeongnam Branch Institute, Korea Institute of Toxicology, Jinju, 52834 Republic of Korea

**Keywords:** Benzyl benzoate, *Danio rerio*, Zebrafish, Proteomics, Label-free proteomic analysis, Biomarker

## Abstract

Benzyl benzoate (BB) is widely used in the food, cosmetics, agriculture, and pharmaceutical industries and is discharged into the aquatic environment via various water sources, including wastewater. Research on the bioaccumulation and possible toxicity of BB has been conducted, but the biochemical responses to BB toxicity are not fully understood, and the specific molecular pathways by which BB causes toxicity remain unknown. In this study, label-free quantitative proteomics based on mass spectrometry was applied to investigate protein profiles in zebrafish (*Danio rerio*) embryos exposed to BB (1 µg/mL) for 7 days. A total of 83 differentially expressed proteins (DEPs) were identified, including 49 up-regulated and 34 down-regulated proteins. The biological functions of proteins regulated by BB were grouped into functional categories and subcategories, including the biosynthesis of organonitrogen compound biosynthetic process, translation, amide biosynthetic process, lipid transport, stress response, and cytoskeletal activity. The results provide novel insight into the molecular basis of the ecotoxicity of BB in aquatic ecosystems.

**Supplementary Information:**

The online version contains supplementary material available at 10.1007/s11356-022-24081-7.

## Introduction

Progress in the chemical industry over the past century has introduced a vast number of chemicals into the environment. Around 100,000 chemicals are in constant use worldwide, and over 500 new chemicals are produced each year (Kim et al. 2018). These chemicals are used in a wide variety of industries, including food, medicine, clothes, and cosmetics, and they are manufactured and consumed in diverse consumer products (Sardar et al. 2019). Concerns about their potential impacts on human health have risen in lockstep with the diversity of chemicals used in consumer products (Hungerbühler et al. 2021). Therefore, toxicity studies are becoming more important because these chemicals can have direct and indirect negative effects on human health, as well as terrestrial and aquatic environments.

Benzyl benzoate (BB) is a naturally occurring substance extracted from aromatic plants (Ramos et al. 2019). BB is an important commercial substance that is used as a dye carrier, plasticizer, and fixative in perfumes, as well as an ingredient in many perfumes, deodorants, and body and sun creams and is discharged into the aquatic environment by various water sources such as wastewater (del Nogal Sánchez et al. 2010; Sriramavaratharajan & Murugan 2018). The amount of BB present in the environment, including soil, wastewater samples, groundwater, and reclaimed water, has not yet been documented in the literature. However, the use of these compounds in high concentrations may result in serious adverse effects such as dermatitis, skin irritation, anaphylactic shock, and seizures (Andaya et al. 2021). BB has also been reported to induce dose-dependent toxicity, including necrosis and transformation of root cells in terrestrial plants (Acar et al. 2020; Diastuti et al. 2020). Several ecotoxicological studies have established that BB is highly toxic to many aquatic organisms; for example, the acute no observable effect concentration (NOEC) is 0.258 mg/L for daphnids and 0.247 mg/L for algae, and the acute LC50 is 2.32 mg/L for fish (Api et al. 2020). Nevertheless, there is limited knowledge about the toxic effects of BB on humans and other animals and the underlying molecular mechanisms.

Recently, multi-OMICs techniques (metabolomics, transcriptomics, and proteomics) have been applied to profile biomarkers in a variety of organisms and to gain a comprehensive and unbiased understanding of the physiological and molecular toxicity of various toxins. (Kwon et al. 2020; Li et al. 2016). In particular, proteomics is a valuable tool for investigating natural disease and chemical toxicity, explaining the molecular events that occur following chemical exposure, and identifying protein biomarkers for chemical pollutants (Chueycham et al. 2021). However, relatively few studies have used proteomics to study BB toxicity in fish species, and to the best of our knowledge, this study is the first to evaluate BB toxicity using a proteomics approach.

The main aim of the present work was to provide a better understanding of the ecotoxicological impacts of BB by evaluating its toxic effects and molecular responses at the protein level in zebrafish (*Danio rerio*) embryos. A label-free quantitative proteomics technique based on mass spectrometry was applied to explore the potential molecular mechanisms of BB exposure. In addition, we evaluated the effects of BB on protein changes in zebrafish by proteome analysis, gene ontology (GO) enrichment, and Kyoto Encyclopedia of Genes and Genomes (KEGG) pathway analysis. The results will aid in the development of potential biomarkers for BB toxicity and help to unveil the associated molecular mechanisms, thereby providing new insights into the ecotoxicity of BB.

## Materials and methods

### Test chemicals

Benzyl benzoate (≥ 99.0% B6630, CAS no. 120–51-4) was purchased from Sigma-Aldrich (St. Louis, MO, USA), and a 50 mg/mL stock solution was prepared in dimethyl sulfoxide (DMSO, Sigma-Aldrich) and diluted to different concentrations with culture medium immediately before use. The same volume of DMSO at a final concentration of 0.1% (v/v) was used as a negative control for zebrafish embryo toxicity tests.

### Zebrafish embryo-larval toxicity tests

Adult zebrafish pairs (AB strain, 6 − 8-month-old) were placed in a mating cage that was designed so that fertilized eggs fell into a space separated from adult fish. Fertilized eggs from 10 adult zebrafish pairs were collected using a 2.0 ± 0.5-mm pore size mesh in a mating cage installed in a spawning trap (width 20 cm, length 22 cm, height 6 cm) and transferred to a glass Petri dish containing tap water filtered through an Advantec 0.45-µm membrane filter (Toyo Roshi Kaisha. Ltd., Japan). Normal fertilized eggs were separated from either unfertilized eggs or abnormal eggs with damaged membranes by microscopy observation using an Olympus IX73 instrument (Olympus, Japan) and used for embryo toxicity tests.

Zebrafish embryo-larval toxicity tests were conducted using sterilized cell culture plates (60 × 15 mm, SPL Life Science, Korea) filled with 15 mL of each test solution, based on standardized toxicity test guidelines for embryo-larval survival and teratogenicity toxicity tests (Park et al. 2020; USEPA 2002). Zebrafish embryos were exposed to six test concentrations of benzyl benzoate ranging from 0 (0.1% DMSO, solvent control) to 12.5 µg/mL for 7 days with four replicates per exposure group (10 embryos per replicate). Test solutions were replaced once every 3 days after exposure. For the BB concentration, one exposure concentration (1 µg/mL) was selected and analyzed by GC–MS/MS (Bruker Daltonics, Billerica, MA, USA) according to the previous study. (Celeiro et al. 2014). No significant changes were detected between nominal concentrations every 2 days (Supplementary Table 1). During the experimental period, the rearing conditions involved a long photoperiod of 16:8 h light/dark at 28 ± 1 °C in a controlled culture system. After BB exposure, lethal (dead + unhatched) and teratogenic larvae were recorded to determine the concentration–response relationship and effective concentration values (EC*x*) for zebrafish embryos.

### Statistical analysis

Zebrafish embryo toxicity was calculated based on USEPA guidelines (2002) using the following formula: Effects (%) = [(mean value for embryo-larval death + teratogenicity including unhatched eggs) / (mean value for initial fertilized eggs) × 100]. EC*x* values and 95% confidence limits for BB were calculated by the probit method (CETIS version 1.8.7.15, Tidepool Scientific Software, USA). Comparison between exposure groups was carried out using the post hoc least squares distance (LSD) one-way analysis of variance (ANOVA) tests (SigmaPlot version 12.5, Systat Software, Inc., San Jose, CA, USA), and *p* < 0.05 was considered statistically significant.

### Protein extraction and sample preparation

For protein extraction, three biological replicates were randomly selected from BB-treated and control groups and subsequently flash-frozen in liquid nitrogen. Zebrafish embryos were homogenized in 1.5-mL pre-chilled Eppendorf tubes in liquid nitrogen. Proteins were extracted in 60 µL of lysis buffer (7 M urea, 2 M thiourea, 4% CHAPS, 1 mM PMSF, 50 mM DTT) via three rounds of sonication on ice using a VCX130 ultrasonic processor (Sonic and Materials Inc., Suffolk, UK) and centrifuged at 10,000 × g for 10 min at 4 °C. The supernatant was collected, and the protein concentration was measured using a 2D-Quant Kit (GE Healthcare Life Sciences, UK) according to the manufacturer’s protocol. Proteins from each sample (100 μg) were then diluted with 2 × Laemmli buffer comprising 65.8 mM Tris–HCl pH 6.8, 26.3% (w/v) glycerol, 2.1% (v/v) SDS, 0.01% (v/v) bromophenol blue, and 2,5% 2-mercaptoethanol (Bio-Rad, CA, USA) and heated for 10 min at 95 °C. Protein samples were run ~ 1.5 cm onto a mini-PROTEAN TGX 12% gel (Bio-Rad) and stained for 20 min using Bio-Safe Coomassie stain (Bio-Rad). Protein-containing areas were cut into five pieces 2 − 3 mm in size, destained in 25% and 50% acetonitrile (ACN) and 25 mM ammonium bicarbonate, and dried using a vc2124 Speed-Vac (Hanil Scientific Inc., Korea). Gel pieces were reduced with 10 mM DTT for 45 min at 56 °C and subsequently alkylated with a 55 mM iodoacetamide for 30 min at room temperature in darkness. For tryptic in-gel digestion, 20 μL of 0.05 μg/mL trypsin (sequencing grade modified trypsin, Promega, Germany) was prepared in 25 mM ammonium bicarbonate and incubated overnight at 37 °C. Digested peptides were acidified with 1% trifluoroacetic acid (TFA) then desalted and purified on Oasis HLB SPE cartridges (30 mg, 1 cc, 30 μm, Waters Corporation, USA) coupled to a vacuum manifold, and samples were reconstituted with 20 μL of 0.1% formic acid (FA).

### Liquid chromatography–tandem mass spectrometry (LC–MS/MS) and protein quantification

Tryptic peptides were analyzed by an Ultimate 3000 RSLC system (Dionex Corp., USA) connected inline to a Q-Exactive Hybrid Quadrupole-Orbitrap mass spectrometer (Thermo Scientific, Bremen, Germany) equipped with an EASY-spray nano-electrospray ion source (Thermo Fisher Scientific) using a 100 min ACN gradient (5–95% ACN). Samples were loaded onto a trapping cartridge (Acclaim PepMap C18 100 Å, 5 mm × 300 µm i.d., 160,454, Thermo Scientific) with a mobile phase of 5% ACN, 0.1% FA, at 3 µL/min. After loading for 5 min, the trap column was switched inline to an ES8000 PepMap RSLC EASY-Spray column (15 cm × 75 μm i.d., C18, 3 μm, Thermo Fisher Scientific) at 300 nL/min. Separation was performed using buffer A (0.1% FA) and buffer B (80% can) with a gradient of 0–5% B over 10 min, 10–40% B over 60 min, 95% B for 15 min, and 95–0% B over 15 min. Data were processed by database searching using Sequest HT (Thermo Fisher Scientific) with Proteome Discoverer 2.3 software (Thermo Fisher Scientific) against the zebrafish (*D. rerio*) section of the UniProtKB database (2017–10-25, 55,761 sequences, 27,742,577 residues) and quantified with a label-free quantification approach. The mass tolerances for precursor and fragment ions were set at 10 ppm and 0.02 Da, respectively. Up to two missed cleavages were permitted using trypsin, and peptides were filtered using a false discovery rate (FDR) of 1% with Benjamini–Hochberg correction.

### Protein classification and functional enrichment analysis

GO annotation and KEGG pathway enrichment analyses of DEPs were performed using GO Resource (http://geneontology.org/) and KOBAS 3.0 (http://kobas.cbi.pku.edu.cn/). DEPs were assigned GO annotations based on their roles in biological process, molecular function, and cellular component categories, using default parameters, and *p* < 0.05 (Benjamini-Hochberg) was considered statistically significant. Protein–protein interaction (PPI) network analysis was conducted to explore the relationships between protein functions of DEPs using the String version 11.0 database (https://string-db.org/), and the network was visualized using Cytoscape 3.9.0 software.

## Results

### Embryo-larval toxicity caused by BB exposure

**Fig. 1:**
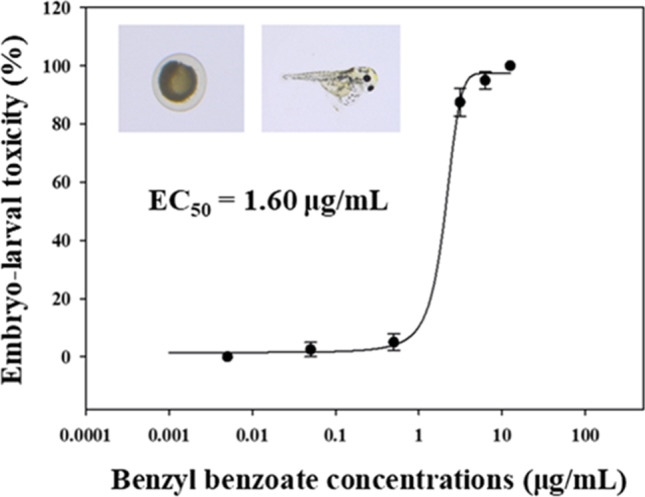
Toxic effects of benzyl benzoate on embryo-larval developmental stages of zebrafish. Fitted sigmoidal dose–response curves were generated for benzyl benzoate. Results are means ± standard error of the mean (SEM) of zebrafish embryo-larval toxicity after 7 days exposure to benzyl benzoate

After BB exposure for 7 days, concentration–response relationships and EC*x* values of zebrafish embryos were determined (Table 1, Fig. 1). The values of EC*x* reveal that higher concentrations of BB may have ecotoxicological effects to morphological defects that eventually result in the death of zebrafish embryos, with concentration–response. However, because the environmental concentrations of BB have not been reported, it is difficult to assess the ecotoxicity of BB based on our results alone. Hatching defects and mortality of embryos were assessed for the 12.5 μg/mL BB-exposed group. There was no significant difference following exposure to > 1 μg/mL of BB compared with the control group, and BB treatment had an EC_50_ value of 1.60 μg/mL (Table 1, Fig. 1). Thus, a proteome study of zebrafish embryos exposed to BB at a concentration of 1 μg/mL (EC_25_) for 7 days was performed to obtain a better understanding of the mechanism by which BB exerts acute toxicity against zebrafish embryos (Table 2).

**Table 1:** Effective concentration values (EC*x*) for benzyl benzoate during embryo-larval developmental stage in zebrafish

Test chemicals	Effective concentrations (μg/mL)		
Benzyl benzoate	EC_10_ (CL)	EC_25_ (CL)	EC_50_ (CL)
	0.60 (0.44–0.71)	0.92 (0.77–1.04)	1.60 (1.36–1.84)

*CL* 95% confidence limit

**Table 2:** Proteins up-regulated in *Danio rerio* embryo exposed to 1 μg/mL benzyl benzoate for 7 days

Protein no	Accession	Protein name	Fold change	Adj. *p*-value
1	Q1LWN2	Vitellogenin 1	3.04	8.8E-15
2	F1R2S5	Vitellogenin 5	4.22	4.1E-16
3	F1RBA0	Vitellogenin 4	5.58	4.1E-16
4	Q1MTC4	Vitellogenin 2	2.08	1.9E-06
5	F1QV15	Vitellogenin 6	4.75	4.1E-16
6	A2BG19	Novel protein similar to vertebrate skeletal alpha-actin 1	1.57	1.7E-02
7	Q1LXJ7	Type I cytokeratin, enveloping layer,-like	> 10	4.1E-16
8	A0A0R4IY49	Vitellogenin 7	2.54	1.7E-10
9	Q5RGY8	Dihydropyrimidinase-like 5b	1.52	3.3E-02
10	Q7ZW04	S-adenosylmethionine synthase	2.12	9.0E-07
11	Q5NJL3	Matrilin-3a	1.68	2.7E-03
12	Q7ZW95	Ribosomal protein L4	1.52	3.3E-02
13	Q6NWL6	Ubiquitin carboxyl-terminal hydrolase	1.52	3.5E-02
14	F1R184	Thioredoxin domain-containing 5	1.63	5.9E-03
15	Q4VBV9	Omega-amidase NIT2	1.53	3.0E-02
16	E7FE19	2′,3′-cyclic nucleotide 3′ phosphodiesterase	1.85	1.3E-04
17	Q58EG2	Erlin-1	7.07	4.1E-16
18	I3ISS5	Ubiquitin-specific peptidase 7 (herpes virus-associated)	1.79	3.9E-04
19	Q6JWU9	Coatomer subunit alpha	1.52	3.5E-02
20	Q0D284	Zgc:153,679	1.73	1.1E-03
21	F1QEX9	Ryanodine receptor 1b (Skeletal)	3.09	4.1E-16
22	F1R3F7	Complement component 1, q subcomponent-binding protein	1.72	1.3E-03
23	Q5BJJ2	Ribosomal protein L3	1.61	8.3E-03
24	Q6NWC4	Gcat protein	1.55	2.1E-02
25	F1QCT0	Mitochondrial import inner membrane translocase subunit TIM50	1.60	1.0E-02
26	Q804W2	Parvalbumin-7	1.85	1.4E-04
27	Q4G5K8	Reticulon	4.66	4.1E-16
28	F1R5M2	Kinesin-like protein	1.71	1.7E-03
29	Q4QRE2	Acyl-coenzyme A oxidase	1.52	3.4E-02
30	Q7T395	Upb1 protein	1.51	3.7E-02
31	Q6P0V6	60S ribosomal protein L8	1.51	3.9E-02
32	Q8QGV4	Lipocalin-type prostaglandin D synthase-like protein	1.78	4.7E-04
33	Q566Z0	LOC553381 protein	> 10	4.1E-16
34	E7FD14	Protein kinase C and casein kinase substrate in neurons 1a	3.86	4.1E-16
35	F1R7F6	UDP-glucose glycoprotein glucosyltransferase 1	> 10	4.1E-16
36	Q7ZWJ6	Zgc:56,466	> 10	4.1E-16
37	Q7ZUW1	Gart protein	1.77	5.4E-04
38	F6P849	Histidine ammonia-lyase	> 10	4.1E-16
39	Q6A3P7	Mothers against decapentaplegic homolog	1.51	3.9E-02
40	Q7ZWA9	Eukaryotic translation initiation factor 5	> 10	4.1E-16
41	Q6PCR6	Zinc finger RNA-binding protein	> 10	4.1E-16
42	Q803T6	SH3 domain-containing GRB2-like 2a, endophilin A1	1.55	2.3E-02
43	F1QXJ4	Syntaxin-binding protein 3	> 10	4.1E-16
44	F1R5X4	Collagen, type XI, alpha 2	3.41	4.1E-16
45	F1RBY1	A kinase (PRKA) anchor protein 1b	> 10	4.1E-16
46	Q6IQT4	COP9 signalosome complex subunit 2	> 10	4.1E-16
47	Q566X7	Npm4 protein	> 10	4.1E-16
48	Q7ZUY0	SET nuclear proto-oncogene a	1.74	1.0E-03
49	E9QEK9	Anterior gradient 1	> 10	4.1E-16

### Proteomic analysis of zebrafish embryo following BB exposure

**Fig. 2:**
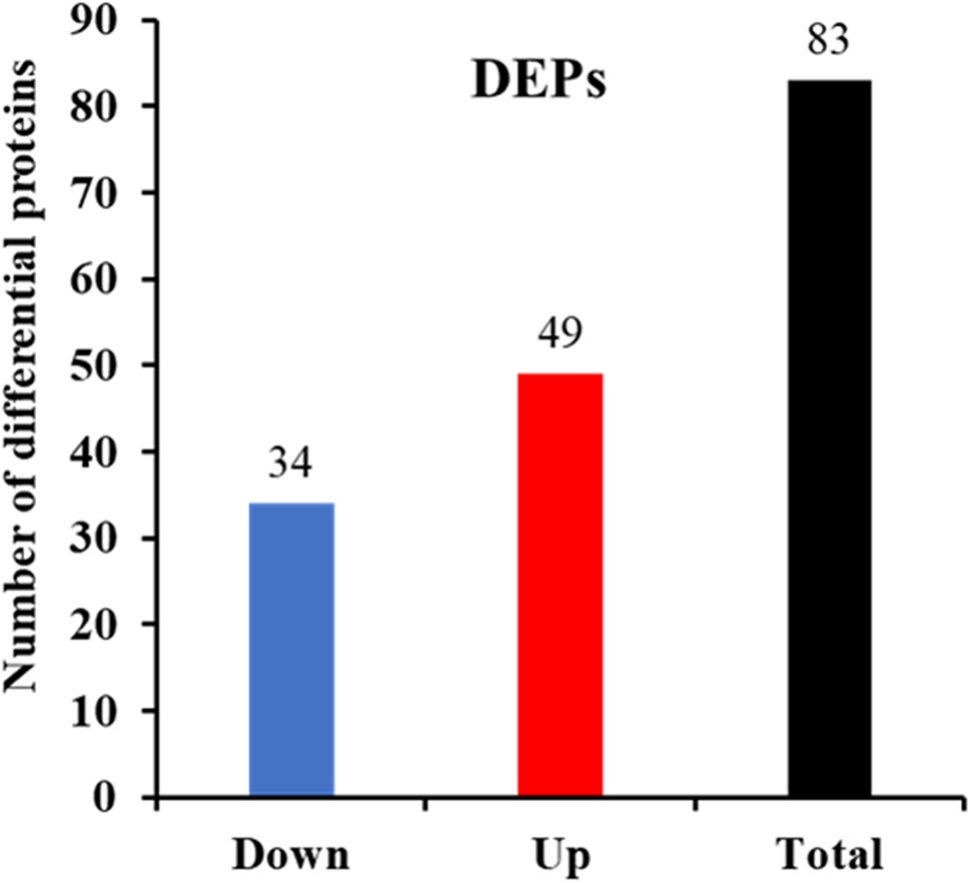
Number of differentially expressed proteins (DEPs) in zebrafish embryos following exposure to benzyl benzoate. Up- and down-regulated proteins are colored blue and red, respectively

**Fig. 3:**
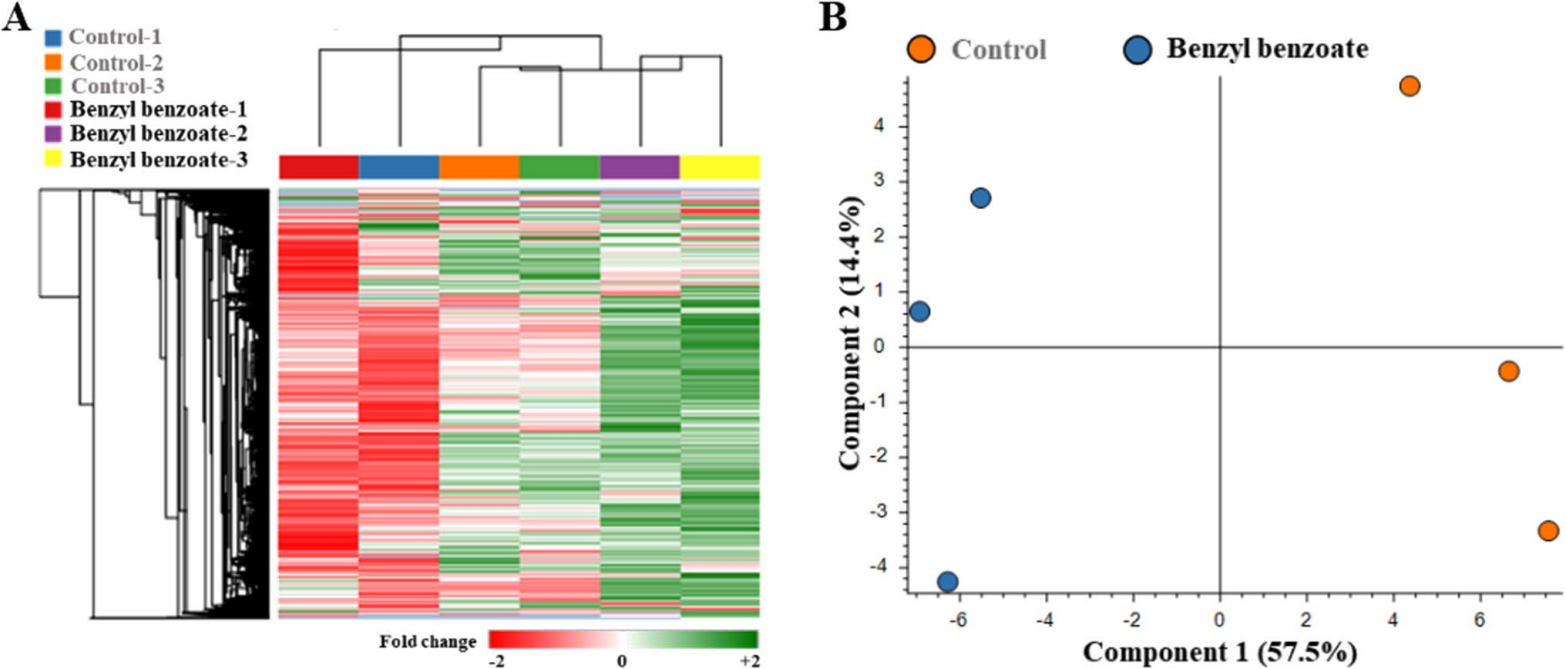
Proteome profiles of *Danio rerio* embryos following 7 days of benzyl benzoate exposure determined by LC–MS/MS analysis. The heatmap shows DEPs in zebrafish embryos following benzyl benzoate exposure based on hierarchical clustering analysis (*p* < 0.05). Columns in the heatmap represent samples, and rows represent distinct proteins. The bar color reflects protein expression levels; green and red indicate up- and down-regulation, respectively. **B** Principal component analysis (PCA) patterns from proteome profiling of control and benzyl benzoate groups based on biological triplicate results

To further study the mechanism by which BB affects zebrafish embryos, we employed label-free quantitative proteome analysis in conjunction with LC–MS/MS. Proteome profiles were determined for zebrafish embryos exposed to 1 µg/mL BB for 7 days (Table 3). A total of 1721 proteins were identified based on 56,304 high-confidence spectra, of which 19,282 peptides were unique. The complete list of identified proteins can be found in Supplementary Table 2. The threshold for screening DEPs was corrected *p* < 0.05 and a ± 1.5-fold change between BB-treated and control groups. This approach led to the identification of 83 significantly altered proteins with FDR < 0.05 between BB-exposed and corresponding control zebrafish embryos (Fig. 2). Compared with the control group, zebrafish embryos treated with BB showed differential protein expression, with 49 up-regulated and 34 down-regulated proteins (Fig. 2). Hierarchical clustering analysis verified the remarkably diverse DEP profiles based on the quantity and distribution of up- and down-regulated (green and red, respectively) proteins, and the results can be visualized using a heatmap (Fig. 3A). Principal component analysis (PCA) showed that samples could be approximately divided using PC1 and PC2 axes, and PC1 accounted for 57.5% of the variance, while PC2 accounted for 14.4% (Fig. 3B).

**Table 3:** Proteins down-regulated in *Danio rerio* embryos exposed to 1 μg/mL benzyl benzoate for 7 days

Protein no	Accession	Protein name	Fold change	Adj. *p*-value
1	F1QK60	Keratin 4	0.551	4.5E-04
2	B0UYS0	Keratin 15	0.01	4.1E-16
3	Q9PV91	Muscle creatine kinase	0.01	4.1E-16
4	A0A0R4IXC8	Tubulin beta chain	0.01	4.1E-16
5	O93548	Embryonic 1 beta-globin	0.658	4.7E-02
6	F6P731	Hemoglobin, alpha embryonic 1.3	0.552	4.7E-04
7	Q6NUT5	Heterogeneous nuclear ribonucleoprotein U-like 1	0.467	1.1E-06
8	Q6NV24	Prostamide/prostaglandin F synthase	0.619	1.2E-02
9	Q90WX5	Cone transducin alpha subunit	0.624	1.5E-02
10	Q6IQQ7	Lumican	0.552	4.7E-04
11	Q7ZV49	Hypoxanthine phosphoribosyltransferase	0.573	1.4E-03
12	Q1JPZ7	Pre-mRNA-processing factor 39	0.426	2.0E-08
13	Q66ID8	Annexin	0.28	4.1E-16
14	E7EZ02	LSM8 homolog, U6 small nuclear RNA-associated	0.546	3.5E-04
15	Q9DDS9	Solute carrier family 3 member 2	0.23	4.1E-16
16	Q568R7	Paralemmin	0.521	7.1E-05
17	Q6Q421	Ribosomal protein S15	0.595	4.2E-03
18	Q5RZ65	Anterior gradient protein 2 homolog	0.01	4.1E-16
19	F1Q9S3	Zgc:66,479	0.531	1.4E-04
20	A0A0R4IDZ0	Erythrocyte membrane protein band 4.1a	0.507	2.6E-05
21	F1R4U6	si:ch211-14a17.10	0.614	9.5E-03
22	B0UYD6	Glutaminyl-tRNA synthetase	0.01	4.1E-16
23	E7F0K3	Caveolae-associated protein 1a	0.01	4.1E-16
24	D6MUD7	Collagen type V alpha-3b	0.575	1.6E-03
25	A5PLK2	Phospholysine phosphohistidine inorganic pyrophosphate phosphatase	0.382	9.3E-11
26	Q6DBU3	LOC553536 protein	0.651	3.7E-02
27	Q6PC37	Nucleoside diphosphate kinase	0.65	3.7E-02
28	Q32Q48	Rbx1 protein	0.01	4.1E-16
29	Q6P3I0	HEAT repeat-containing 3	0.624	1.4E-02
30	A4JYG8	Cntn2	0.101	4.1E-16
31	Q6NY24	Phospholipid scramblase	0.01	4.1E-16
32	E7F520	T-cell lymphoma invasion and metastasis 1b	0.482	3.9E-06
33	Q5RH28	Inter-alpha-trypsin inhibitor heavy chain 2	0.01	4.1E-16
34	Q6P0H6	COP9 signalosome complex subunit 4	0.01	4.1E-16

### Functional analysis of DEPs

**Fig. 4:**
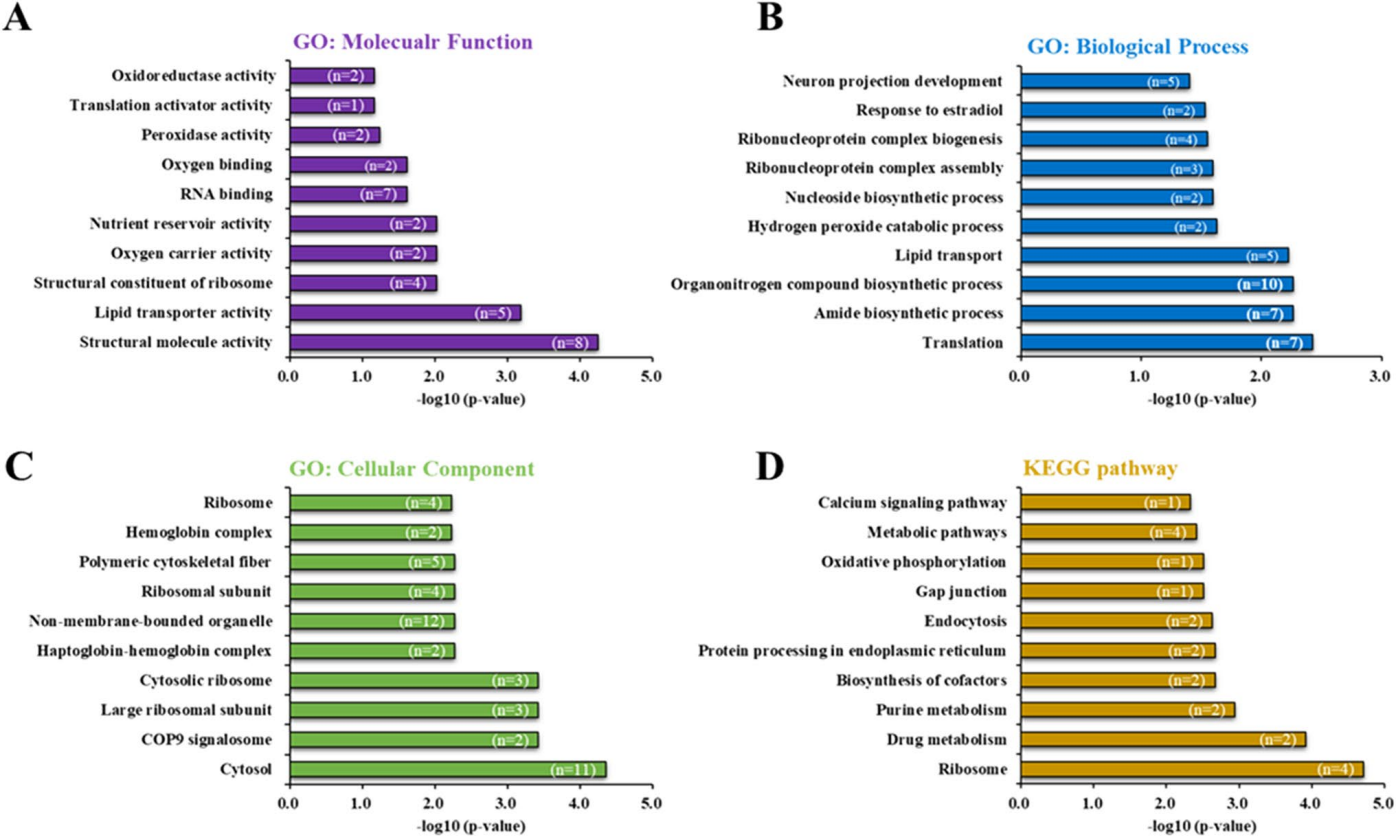
GO enrichment and KEGG pathway analyses of 83 DEPs in zebrafish embryos following benzyl benzoate exposure. **A** Molecular functions. **B** Biological processes. **C** Cellular component. **D** KEGG pathways. Log_10_ (*p*-values) are shown on the x-axis, and the top 10 items from each group are displayed on the y-axis. Only GO terms and KEGG pathways with a *p* < 0.05 were included, and the number of associated proteins is indicated in brackets

We investigated the interactions and functions of 83 DEPs between control and BB-treated groups using KOBAS 3.0 (Xie et al. 2011), in an attempt to unravel the probable physiological changes that contributed to the harmful effects of BB exposure. Protein function classification enabled the identification of biological processes, molecular functions, and cellular components related to BB-induced toxicity. The most enriched subcategories among molecular functions were structural molecule, lipid transporter, RNA binding, and oxidoreductase activity (Fig. 4A). The most enriched subcategories among biological processes were organonitrogen compound biosynthetic process, translation, amide biosynthetic process, and lipid transport (Fig. 4B). The most enriched subcategories among cellular components were cytosol, non-membrane-bounded organelle, ribosome, and COP9 signalosome (Fig. 4C). KEGG pathway analysis of the identified proteins showed that most were associated with metabolic pathways, ribosomes, drug metabolism, purine metabolism, endocytosis, and calcium signaling (Fig. 4D).

**Fig. 5:**
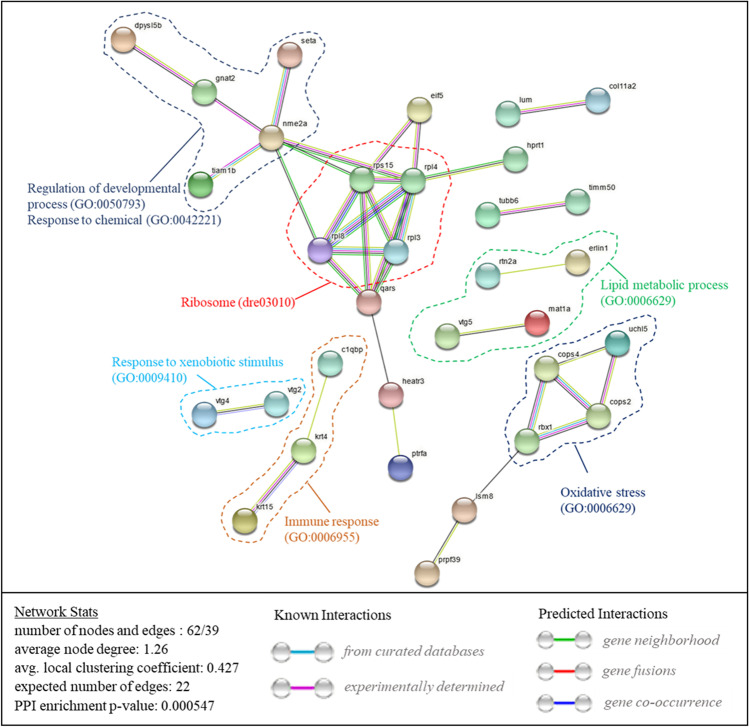
Protein–protein interaction (PPI) regulatory network of differentially regulated proteins in *Danio rerio* embryos after exposure to benzyl benzoate. Nodes indicate proteins, while edges represent protein–protein associations. Model statistics are displayed in the bottom left corner, and edge color explanations are displayed in the lower right. Protein clusters associated with certain biological processes or metabolic routes are denoted by dashed lines

In addition, to better understand the roles of DEPs, a PPI network was constructed using the STRING database (v11.5). The DEPs formed a complex interaction network with 62 nodes and 39 edges, with an average node degree of 1.26, and a clustering coefficient of 0.427. The expected number of edges was 22, which was substantially lower than the actual number of edges, and the *p*-value for PPI enrichment was 5.5e-04 (Fig. 5). PPI network analysis showed that exposure to BB primarily affected metabolic processes and ribosomes in zebrafish embryos. Other affected processes included regulation of developmental processes, responses to xenobiotic stimuli, lipid metabolic processes, oxidative stress, and immune responses. Thus, functional analysis of DEPs indicated that the potential toxicity of BB against zebrafish was likely mediated via several diverse physiological processes.

## Discussion

Zebrafish is an excellent species for proteomic, genomic, and transcriptomic analyses because it is a well-characterized vertebrate animal model widely used in genetics, physiology, and immunology (Lü et al. 2014; Sullivan & Kim 2008). The zebrafish genome has been completely sequenced and largely annotated, which makes it simpler to discover and describe proteins using proteomic analysis in conjunction with existing databases (Molina et al. 2021; Toni et al. 2019). Furthermore, proteomics data collected on zebrafish can serve as a valuable resource for future research into protein networks and biological evolution. However, few studies have investigated the molecular pathways associated with BB exposure in zebrafish embryos. Herein, we present the results of a proteome analysis of BB-exposed zebrafish embryos and discuss the host responses to this potentially harmful chemical. To our knowledge, this is the first label-free quantitative proteomics study on the effects of BB in zebrafish embryos. We identified numerous differentially regulated proteins in BB-treated embryos, with 34 and 49 significantly down- and up-regulated compared to controls, respectively, indicating that BB affects the overall embryonic proteome.

Protein synthesis and its associated machinery plays a pivotal role in regulating cell proliferation and metabolism and maintaining homeostasis via regulation of gene expression under abiotic stress conditions to support the translation process (Liu et al. 2015). It is known that some species regulate ribosomal proteins in response to diverse environmental stresses and that the synthesis and conversion of huge numbers of proteins occurs as a result of these responses (Zaucker et al. 2020). Chlorpyrifos causes ribosome damage in zebrafish, observed as a gradual decrease in the production of new proteins (Liu et al. 2015). By contrast, the toxic effects of methyl parathion and cadmium, in combination, impact the de novo synthesis of ribosomal proteins (Ling et al. 2012). In the present study, ribosomal proteins RPl4, RPl3, and RPl8 were up-regulated, while RPS15 was down-regulated, suggesting that BB may affect the growth of zebrafish embryos and trigger apoptosis through impacts on protein translation and synthesis.

Exposure to BB induced the down-regulation of embryonic 1 beta-globin (HBBE1.3), a protein associated with oxygen transport and cellular detoxification. HBBE1.3 is encoded by five genes located on the short region of chromosome 11 and is responsible for the production of hemoglobulin beta, which is crucial for oxygen transport (de Queiroz et al. 2021). Regulation of HBBE1.3 in aquatic fish is altered by exposure to a wide range of environmental pollutants (Della Torre et al. 2018; Eissa & Wang 2016; Hernández et al. 2021), as well as changes in environmental stress conditions such as temperature, salinity, and hypoxia (Duarte et al. 2010; Narra 2016). Down-regulation of HBBE1.3 suggests that BB exposure might damage embryo development and survival, as well as cellular detoxification, by limiting oxygen delivery to cells.

Collagen type XI alpha 2 (COLLa2), tubulin beta chain (TUBB6), Type I cytokeratin (CYT1L), and two keratin proteins (KRT4 and KRT15) were differentially regulated by BB. Cytoskeleton proteins are involved in a variety of physiological activities, including cell motility, muscle contraction, and cytokinesis (Fletcher & Mullins 2010; Rocha et al. 2015; Xu et al. 2019a, b). Several studies have demonstrated that oxidative and chemical stresses can cause alterations in cytoskeletal proteins. Thus, the considerable decrease in KRT15, TUBB6, and KRT4 expression observed in this study could be a result of BB-induced toxicity and oxidative stress (Fu et al. 2018; Wang et al. 2020; Xu et al. 2019a, b; Yan et al. 2020). Previous proteomics research found that environmental pollutants such as heavy metals can cause cytoskeletal damage in marine bivalves and that tubulins, actins, and keratins are often involved in cytoskeletal injuries (Wu et al. 2013; Xu et al. 2016). Therefore, changes in these five cytoskeleton-associated proteins may cause cytoskeletal damage and structural impairment zebrafish in following BB exposure.

In this study, we discovered that six vitellogenin (VTG) proteins (VTG1, VTG2, VTG4, VTG5, VTG6, and VTG7) were significantly up-regulated in *D. rerio* exposed to BB, according to label-free LC–MS/MS proteomic analysis. VTGs are phospholipid glycoproteins present in non-mammalian species that promote the growth and differentiation of oocytes and provide nutrients for embryonic development (Liu et al. 2018; Meng et al. 2019). Toxic substances in the environment can have negative effects on a variety of processes, including the deposition of maternal yolk, by interfering with the production of VTGs (Hanisch et al. 2020; Sant & Timme-Laragy 2018). For this reason, VTGs have been employed as sensitive biomarkers for hazardous substances, particularly endocrine-disrupting chemicals (EDCs), in the aquatic environment (He et al. 2019; Zhang et al. 2019). Several previous studies have shown that VTG accumulation occurs following exposure to toxic substances and EDCs, including endosulfan, phthalates, bisphenol A, tetrabromobisphenol A, and 17α-ethinylestradiol (Chow et al. 2013; Kausch et al. 2008; Keiter et al. 2012; Örn et al. 2016; Uren-Webster et al. 2010). Given that the induction of VTGs is a biomarker for exposure to environmental contaminants such as estrogen-active chemicals, the increased expression of VTGs in our proteomic analysis suggests that the toxicity of BB in fish is likely to mimic the reproductive toxicity of EDCs.

Phospholipid scramblases (PLSCRs), encoded by a tetrad of genes widely conserved in species from *Caenorhabditis elegans* to humans, are a structurally and functionally distinct class of proteins (Rayala et al. 2014). PLSCR3 is involved in the translocation of phospholipids between mitochondrial lipid compartments and plays a role in the morphology, function, and apoptotic responses of mitochondria (de la Ballina et al. 2020). In response to stress signals, *plscr3* is phosphorylated by mitochondrial translocation of protein kinase C delta, which is activated during apoptosis and plays a role in rearranging phospholipids within the mitochondrial membrane, thereby mediating the apoptotic response (Sivagnanam et al. 2017). Moreover, down-regulation of *plscr3* promotes resistance to the effects of apoptosis (Arashiki & Takakuwa 2017). Our proteomic analysis showed that expression levels of PLSCR3 were decreased following exposure to BB.

The COP9 signalosome (COPS) is an important protein complex that inhibits protein degradation, and it has been linked to the maintenance of pluripotency (Chia et al. 2010). COPS subunit 2 (*cops2*) is a critical component, found in both the cytoplasm and the nucleus, and a highly conserved multiprotein complex involved in cellular and developmental processes (Koyuncu et al. 2015; Zhang et al. 2016). COPS2 serves as a negative regulator of nucleic acid-templated transcription and protein deneddylation (Li et al. 2018). In the present study, COPS2 was found to be up-regulated in the BB-exposed group compared to the control group. The identification of biomarkers for hazardous compounds such as BB is critical for environmental chemical analysis. However, the role of COPS2 in the proteome responses to toxic impacts on aquatic animals remains unknown, but it may play a role in the direct toxic effects of BB on zebrafish embryos. Nonetheless, further research is required to elucidate the functions and protein interactions under chemical stress conditions.

In conclusion, our findings contribute to a better understanding of BB toxicity in zebrafish, which is a significant step forward in the field. The EC_50_ value of BB against zebrafish embryos was 1.60 g/mL, and hatching defects and mortality of embryos were observed at a BB concentration of 12.5 g/mL. Proteomic analysis revealed that BB had a significant effect on proteins involved in a variety of processes, including the biosynthesis of organonitrogen compounds, translation, amide biosynthesis, lipid transport, stress responses, and cytoskeletal activity. To the best of our knowledge, this is the first study to use proteomics to examine the ecotoxicological effects of BB in fish, and the potential biomarkers discovered will aid in the assessment of BB contamination and toxicity in aquatic environments.

## Supplementary Information

Below is the link to the electronic supplementary material.

Supplementary file1 (DOCX 21 KB)

Supplementary file2 (XLSX 342 KB)

## Data Availability

On reasonable request, the corresponding author will provide the datasets used and/or analyzed during the current work.
