# Supplementary material for: Proteomic analysis of zebrafish (Danio rerio) embryos exposed to benzyl benzoate
Source: Environ Sci Pollut Res Int. 2022 Nov 11;30(10):26375–86. doi: 10.1007/s11356-022-24081-7 (PMC9995408; doi:10.1007/s11356-022-24081-7)
Supplement: Supplementary file 1 — Supplementary file1 (DOCX 21 KB) [file 11356_2022_24081_MOESM1_ESM.docx]

**Supplementary Table 1. The concentration of benzyl benzoate in exposure solutions using GC-MS/MS analysis (n = 3)**

| Exposure period | Nominal concentration (μg/mL) | Calculated concentration (μg/mL) |
| --- | --- | --- |
| 2 days | 1 | 1.14 ± 0.12 |
| 4 days | 1 | 1.06 ± 0.05 |
| 6 days | 1 | 0.97 ± 0.14 |

The measured concentrations were expressed as mean ± standard deviation.
